# Sensitive Indicators of Zonal *Stipa* Species to Changing Temperature and Precipitation in Inner Mongolia Grassland, China

**DOI:** 10.3389/fpls.2016.00073

**Published:** 2016-02-08

**Authors:** Xiaomin Lv, Guangsheng Zhou, Yuhui Wang, Xiliang Song

**Affiliations:** ^1^State Key Laboratory of Vegetation and Environmental Change, Institute of Botany, Chinese Academy of SciencesBeijing, China; ^2^Department of Life Science, University of Chinese Academy of SciencesBeijing, China; ^3^Chinese Academy of Meteorological SciencesBeijing, China

**Keywords:** *Stipa* species, precipitation changes, warming, sensitive indicator, Inner Mongolia grassland

## Abstract

Climate change often induces shifts in plant functional traits. However, knowledge related to sensitivity of different functional traits and sensitive indicator representing plant growth under hydrothermal change remains unclear. Inner Mongolia grassland is predicted to be one of the terrestrial ecosystems which are most vulnerable to climate change. In this study, we analyzed the response of four zonal *Stipa* species (*S. baicalensis, S. grandis, S. breviflora*, and *S. bungeana*) from Inner Mongolia grassland to changing temperature (control, increased 1.5, 2, 4, and 6°C), precipitation (decreased 30 and 15%, control, increased 15 and 30%) and their combined effects via climate control chambers. The relative change of functional traits in the unit of temperature and precipitation change was regarded as sensitivity coefficient and sensitive indicators were examined by pathway analysis. We found that sensitivity of the four *Stipa* species to changing temperature and precipitation could be ranked as follows: *S. bungeana* > *S. grandis* > *S. breviflora* > *S. baicalensis*. In particular, changes in leaf area, specific leaf area and root/shoot ratio could account for 86% of the changes in plant biomass in the four *Stipa* species. Also these three measurements were more sensitive to hydrothermal changes than the other functional traits. These three functional indicators reflected the combination of plant production capacity (leaf area), adaptive strategy (root/shoot ratio), instantaneous environmental effects (specific leaf area), and cumulative environmental effects (leaf area and root/shoot ratio). Thus, leaf area, specific leaf area and root/shoot ratio were chosen as sensitive indicators in response to changing temperature and precipitation for *Stipa* species. These results could provide the basis for predicting the influence of climate change on Inner Mongolia grassland based on the magnitude of changes in sensitive indicators.

## Introduction

Global warming that is caused by increased concentrations of CO_2_ and other greenhouse gases is likely to increase global mean temperature by 1.0–3.7°C at the end of this century based on the most recent projections (IPCC, 2013). Meanwhile, global warming is projected to shift precipitation patterns and exacerbate extreme climate events (Tramblay et al., 2012). Consequently, plant growth, distribution, adaptive strategy and productivity (Sherry et al., 2008; Xu et al., 2013, 2014; Maréchaux et al., 2015), even the structure and function of ecosystem (Bai et al., 2004; Albert et al., 2011; Craine et al., 2012; Wilcox et al., 2015), have been dramatically affected by global warming. Therefore, taking adaptive countermeasures has become a global focus (IPCC, 2013; Palumbi et al., 2014). But if we want to scientifically implement adaptive strategies, we must firstly understand the effects of global warming on plants at every level (physiological ecology, structure, and function, etc.), and then determine how global warming affects plants. Meanwhile we urgently need to examine sensitivity degree of different plants to climate change and determine how to create and use a sensitivity index reflecting whole plant growth (Brunner et al., 2015). These analyses could provide a scientific basis for quantitatively evaluating the responses of terrestrial ecosystems to climate change and to mitigating that change effectively.

Temperature and precipitation are important climatic factors related to plant growth, but changes in those factors result in various effects on many plant functional traits (Xu et al., 2013; Springate and Kover, 2014; Maréchaux et al., 2015). In general, changes in temperature and precipitation mainly affect plants' production capacity and adaptive strategies. For example, moderate warming could enhance plants' production capacity, including plant development and biomass accumulation (Wu et al., 2011; Xu et al., 2013). However, warming also suppresses total production and root production of some herbaceous plants (Hoeppner and Dukes, 2012) or has little effect on plant biomass of *Caragana microphylla* (Xu et al., 2014). Mild drought has no significant effect on plant growth, but extreme drought would reduce plant height (PH), leaf area (LA) or photosynthetic ability, and even inhibits a plant's production capacity (Mumson et al., 2013; Sapeta et al., 2013). Additionally, a plant often shifts its adaptive strategies in response to the limitations produced by temperature or precipitation to maintain growth (Yang et al., 2010; Comas et al., 2013). For example, early-blooming plant species could prolong their growing season under night warming in semi-arid grasslands (Xia and Wan, 2012), some plants increase their root/shoot ratio (R/S) in response to decreased temperatures in Inner Mongolian grassland (Fan et al., 2009), and some plants also increase the ratio of root biomass or decreased the ratio of leaf and stem biomass to allocate more resources to roots under water deficit conditions (Yin et al., 2005; Guo et al., 2007). Increases in root/leaf ratio and specific leaf area (SLA) were also found in *Populus tremula* and *Caragana microphylla* to maintain its individual moisture balance and individual growth, especially under extreme drought (Yin et al., 2005; Xu et al., 2014).

However, the combined effects of temperature and precipitation on a plant are different from their individual effects. Numerous studies have found that sensitivity of plants to high temperature could be improved, and their tolerance to warming stress would be reduced by drought (Xu and Zhou, 2006; Hoeppner and Dukes, 2012). For instance, warming benefits the growth of herbaceous plants, especially *leguminous* plants, but seasonal variations in rainfall amount could decrease sensitivity of herbs to warming (Hopkins and Del Prado, 2007). Nighttime warming could result in a significant decrease in plant biomass, LA and SLA of *Leymus chinensis*; however, these adverse effects are strengthened by water deficits (Xu and Zhou, 2006). These previous studies have elucidated the effects of climate change on a large number of plant functional traits (Sherry et al., 2008; Hoeppner and Dukes, 2012), but relationship of the changes in various functional traits and which trait is relatively more vulnerable in response to climate change are neglected. Several studies have reported that plant leaves were sensitive to environmental change because leaf is the main place where photosynthesis and respiration occur (Catoni and Gratani, 2014; Salgado-Negret et al., 2015). Similarly, plants mainly shifted resource allocation to roots to maintain the individual growth ratio under drought and warming conditions (Bret-Harte et al., 2001; Xu et al., 2014). So we can see that plant leaf and root mass partitioning might respond significantly to climate change to a certain degree, but quantitative analysis for the degree of sensitivity of these functional traits in response to temperature and precipitation remains unknown. Currently only a limited suite of countermeasures are available to develop adaptive strategies of plant species as they relate to climate change. As a consequence, a better understanding of which trait is more sensitive and indicates the response of whole plant growth to climate change can provide new insights into how plants adapt to a changing world.

The temperate grasslands of Inner Mongolia, China are an important part of the Eurasian grassland biome. These grasslands support diverse plant and animal species and play important roles in providing ecological services and socio-economic development of the region (Kang et al., 2007). Temperatures have increased substantially in this region during the past 50 years (Wan et al., 2009) and summer precipitation is predicted to increase or decrease in different grassland types (Cholaw et al., 2003). Recently numerous studies also indicated that dominant plant species in Inner Mongolia have faced species alteration and degradation in recent years (Luo et al., 2012; Zhang et al., 2013). Therefore, this region has already been documented as being very sensitive and relatively fragile to global climate change (Niu and Wan, 2008; Sui et al., 2013). *Stipa* species serve as the dominant plants of zonal grassland communities and present an ecological alternating distribution along a changing precipitation gradient from east to west in Inner Mongolia. This distribution reflects local plant-environment interactions that demonstrate the various adaptabilities of *Stipa* species to environmental change over a long evolutionary time scale. Of these species, *S. baicalensis, S. grandis, S. breviflora*, and *S. bungeana* are the dominant species in the arid and semiarid grassland of Inner Mongolia and are mainly distributed in meadow steppe, typical steppe, desert steppe and warm-temperate typical steppe, respectively (Qi et al., 2010; Hu et al., 2013, 2014). These four *Stipa* species distribute in various regions in Inner Mongolia grassland with different demand of temperature and precipitation, their sensitivity degree to hydrothermal changes are not well understood.

Plant functional traits represent the internal or external characteristics that plants adapt to and interact with the environment. Changes in plant functional traits can express the sensitivity of plants and ecosystem in response to the external environment (Craine et al., 2002; de Bello et al., 2010; Salgado-Negret et al., 2015). Several studies have also indicated that function and structure of grassland ecosystems are more susceptible to the responses of dominant plants to climate change (Hou et al., 2013; Xu et al., 2014; Yuan et al., 2015). Therefore, using climate control chambers, the main objective of this study was to examine sensitivity of different plant functional traits in response to temperature and precipitation change. This study analyzed those traits in four dominant *Stipa* species (*S. baicalensis, S. grandis, S. breviflora*, and *S. bungeana*) from the grasslands of Inner Mongolia. There are three hypotheses were tested: (1) as decrease in water can strengthen the adverse effects of warming to some plant growth (Xu and Zhou, 2006; Hopkins and Del Prado, 2007; Hoeppner and Dukes, 2012), the sensitivity of plant functional traits in response to temperature may be enhanced by changes in precipitation to a certain degree, and vice versa; (2) Previous studies have shown that *S. baicalensis* is dominant species in arid and semiarid grassland of Inner Mongolia and mainly distributes in meadow steppe with sufficient precipitation and stable growth, *S. grandis* and *S. breviflora* respectively distribute in typical steppe and desert steppe with developed root system, *S. bungeana* is a constructive species in warm-temperate typical steppe with a relative scarce of precipitation and a warm climate and has a higher demand of the cooperative effects of hydrothermal factors (Qi et al., 2010; Hu et al., 2013, 2014). So among the sensitivity of the four *Stipa* species to hydrothermal changes, *S. bungeana* may be the most sensitive species, followed by *S. breviflora* and *S. grandis*, and *S. baicalensis* was the least sensitive species; (3) Plant leaves are sensitive to environmental change as leaf is the main place where photosynthesis and respiration occur (Catoni and Gratani, 2014; Salgado-Negret et al., 2015), and plants primarily maintain individual growth via altering root mass partition under drought and warming conditions (Bret-Harte et al., 2001; Xu et al., 2014), so LA and R/S of *Stipa* species may be more sensitive indicators among functional traits to combined effects of temperature and precipitation in this study.

## Materials and methods

### Plant culture

Seedlings of *Stipa* species were used to study their functional traits in response to anticipated climate change. During the year prior to the experiment, seeds were collected from local steppe habitats in Inner Mongolia, China. Seeds of *S. baicalensis, S. grandis, S. breviflora*, and *S. bungeana* were collected from a natural meadow steppe in Hulun Buir (49°13′N, 119°45′E), a typical steppe in Xilinhot (43°38′N, 116°42′E), a desert steppe in Siziwang Banner (41°43′N, 111°52′E) and a sandy grassland in Ordos (39°50′N, 109°59′E), respectively (Table 1). Each *Stipa* plant was the dominant species of each respective site. Seeds from each species were sterilized in a 5% potassium permanganate solution for 8 min and then rinsed before sowing. Sowing was performed in plastic pots (10.9 cm in diameter, 9.5 cm in height, and 0.71 L in volume) with plastic film on the bottom as a seepage control measure. Each plastic pot was filled with 0.58 kg of air-dried soil that was retrieved from the local surface soil (0–30 cm) of the four corresponding plants. All pots were initially placed in a naturally illuminated glasshouse (day/night temperature of 26–28°C/18–20°C, maximum photosynthetic photon flux density of 1000 μmol·m^−2^.s^−1^) until the third leaf emerged (3 weeks after sowing). Then, the plants were transferred into climate control chambers (RXZ-500D, The Southeast Instruments Inc., Ningbo, China) and subjected to a respective 3-month warming and precipitation change treatment as described below.

**Table 1:** **The geographical information related to seed collections for the four ***Stipa*** species with average temperature and precipitation data from June to August during 1978–2007 (30 years)**.

**Species**	**Collecting regions**	**Geolocation**	**Elevation (m)**	**Average temperature (°C)**	**Average precipitation (mm)**
				**June/July/August**	**June/July/August**
*S. baicalensis*	Hulun Buir	49°13′N, 119°45′E	628–649	17.9/20.3/18.2	55/94/90
*S. grandis*	Xilinhot	43°57′N, 116°07′E	1265–1300	19.1/21.4/19.6	45/78/65
*S. breviflora*	Siziwang Banner	41°43′N, 111°52′E	1420–1500	17.9/20.4/18.0	41/77/81
*S. bungeana*	Ordos	39°50′N, 109°59′E	1350–1400	19.2/21.3/19.3	51/97/99

### Experimental design

The warming and precipitation change experiments were conducted at the greenhouse in the Institute of Botany, Chinese Academy Sciences, Beijing, China. Thirty-year (1978–2007) monthly average temperature and precipitation of the growing season (June, July, and August) for the four sites were used as baselines (Table 1). Based on future climate change scenarios, two factors were designed in this experiment: five temperature patterns including T_0_ (normal temperature, local monthly average temperature), T_1.5_ ($T_{0}+1.5^{{}^{\circ}}$C), T_2.0_ ($T_{0}+2^{{}^{\circ}}$C), T_4.0_ ($T_{0}+4^{{}^{\circ}}$C), and T_6.0_ ($T_{0}+6^{{}^{\circ}}$C); and five precipitation patterns, including W_0_ (normal precipitation, local monthly average precipitation), W_−30%_ (W_0_−30%), W_−15%_ (W_0_−15%), W_+15%_ (W_0_+15%), and W_+30%_ (W_0_+30%). For each species, five climate control chambers were used for five temperature treatments and with separate precipitation treatments within each chamber as a split plot. That is, for each temperature treatment, five different irrigation treatments were used with six replicates (six pots, each with four plants) for each species. This created 150 plots in total for each species. Precipitation was added to the pots by a sprayer at approximately 17:00 every 3 days, as described in our previous similar experiments (Xu and Zhou, 2006; Xu et al., 2014). The pots were randomly placed into each chamber, and moved stochastically within each layer (exchanging pot positions from center to edge and vice versa) every 3 days and between two layers every week to avoid the effects of an uneven distribution of environmental conditions. The pots were also transferred between the five chambers every 2 weeks to minimize any difference between growth chambers, except for desired treatments of temperature and water regimes (Qaderi et al., 2012; Xu et al., 2014).

### Plant harvest

At the end of the experiment, three categories of functional traits (morphological characteristics: PH, the number of leaves (LN), LA, biomass: aboveground biomass (AB), belowground biomass (BB), total biomass (TB), and growth index: SLA, leaf mass ratio (LAR), R/S) of each species under each treatment were measured. PH and LN of each plant were determined prior to harvest. Harvested plants were further separated into stems, roots, green leaves and dead leaves. LA per plant was measured with a WinFOLIA system for root/leaf analysis (WinRhizo, Régent Instruments, Quebec, Canada). Separated items were then oven-dried at 80°C to a constant weight to obtain biomass. AB summed from the biomass of stems, green leaves and dead leaves was added to BB to obtain total biomass. Three replicates of LA in one temperature and precipitation treatment were used to measure other physiological indices, so only three replicates of biomass within one treatment were obtained. The following plant growth indices were calculated according to Poorter (1999). SLA (m^2^·kg^−1^), LAR (m^2^·kg^−1^), and R/S were expressed as leaf area/its mass, leaf area/plant total biomass, root biomass/aboveground biomass, respectively.

### Sensitivity analysis

Sensitivity refers to the degree of response of a system to climate change; however, this response may be favorable or detrimental (IPCC, 2013). If *y* ecosystem is a function in a variety of exposure *x*_*i*_, *y* = *f* (*x*_1_, *x*_2_,…, *x*_*n*_) (*x*_*i*_ is the attribute value of *y* in the *i*). The degree of sensitivity is generally regarded as the relative change of *y* ecosystem under the change of exposure *x*_*i*_ within a certain range (Luers, 2005; Cariboni et al., 2007; Cui et al., 2009). Therefore, the sensitivity coefficient can be expressed as Δ*y*/Δ*x*_*i*_. In this study, compared to control temperature and precipitation treatment, the sensitivity of plant functional traits to changing temperature and precipitation could be regarded as the relative change of the functional traits in the unit of temperature and precipitation change. The greater the absolute value of the sensitivity coefficient, the greater the sensitivity of functional trait is to hydrothermal change. The sensitivity coefficient (*SC*) to changing temperature or precipitation is expressed as:

(1) $$\begin{array}{rcl} {SC}_{jm} & = & \frac{1}{n}\sum_{i=1}^{n}{SC}_{ijm} \end{array}$$

(2) $$\begin{array}{rcl} {SC}_{ijm} & = & △y_{ijm}∕△x_{im} \end{array}$$

(3) $$\begin{array}{rcl} △y_{ijm} & = & \left(y_{ijm}-y_{0jm}\right)∕y_{0jm}\times 100\% \end{array}$$

(4) $$\begin{array}{rcl} △x_{im} & = & x_{im}-x_{0m} \end{array}$$

(5) $$\begin{array}{rcl} {SC}_{m} & = & \frac{1}{r}\sum_{j=1}^{r}{SC}_{jm} \end{array}$$

Where *i* is the various temperature or precipitation treatments; *j* is the various functional traits; *r* is the number of functional traits, where *r* = 9; *m* is different plant species; *n* is the number of temperature or precipitation treatment except for control treatment, where *n* = 4; Δ*x*_*im*_ is the variation of temperature or precipitation in treatment *i* for species *m*; *x*_*im*_ is the value of temperature or precipitation in treatment *i for* species *m*; *x*_0*m*_ is the value of temperature or precipitation in the control treatment for species *m*; Δ*y*_*ijm*_ is the relative variation of functional trait *j* under treatment *i* for species *m*; *y*_*ijm*_ is the value of functional trait *j* under treatment *i* for species *m*; *y*_0*jm*_ is the value of functional trait *j* under the control treatment for species *m*. *SC*_*ijm*_ is the sensitivity of functional trait *j* to temperature or precipitation treatment *i* for species *m*; and *SC*_*jm*_ is the sensitivity of functional trait *j* for species *m, SC*_*m*_ is the sensitivity of species *m*.

Similarly, *SC* to combined effects of temperature and precipitation is expressed as:

(6) $$\begin{array}{rcl} {SC^{\prime }}_{jm} & = & \frac{1}{n}\sum_{i=1}^{n}{SC^{\prime }}_{ijm} \end{array}$$

(7) $$\begin{array}{rcl} {SC^{\prime }}_{ijm} & = & △{y^{\prime }}_{ijm}∕△g_{im} \end{array}$$

(8) $$\begin{array}{rcl} △{y^{\prime }}_{ijm} & = & \left({y^{\prime }}_{ijm}-{y^{\prime }}_{0jm}\right)∕y_{0jm}\times 100\% \end{array}$$

(9) $$\begin{array}{rcl} △g_{im} & = & \left(T_{im}-T_{0m}\right)\times \left(P_{im}-P_{0m}\right) \end{array}$$

Where *i* is the various combined temperature and precipitation treatments; *j* is the various functional traits; *m* is different plant species; *n* is the number of combined temperature and precipitation treatment except for control treatment, where *n* = 16; Δ*g*_*im*_ is the variation of combined temperature and precipitation in treatment *i* for species *m*; *T*_*im*_ is temperature in treatment *i* for species *m*; *T*_0*m*_ is temperature in the control treatment for species *m*; *P*_*im*_ is precipitation in treatment *i* for species *m*; *P*_0*m*_ is precipitation in control treatment for species *m*; Δ*y*′_*ijm*_ is the relative variation of functional trait *j* under treatment *i* for species *m*; *y*′_*ijm*_ is the value of functional trait *j* under treatment *i* for species *m*; *y*′_0*jm*_ is the value of functional trait *j* under control combined temperature and precipitation treatment (T_0_W_0_) for species *m*. *SC*′_*ijm*_ is the sensitivity of functional trait *j* to combined temperature and precipitation treatment *i* for species *m*; and *SC*′_*jm*_ is the sensitivity of functional trait *j* to combined effects of temperature and precipitation for species *m*.

The average *SC* of morphological characteristics to combined effects of temperature and precipitation is computed as the average *SC* of PH, LN, and LA. Likewise, the average *SC* of biomass and growth index to combined effects of temperature and precipitation are respectively determined as the average *SC* of biomass (AB, BB, TB) and growth indices (SLA, LAR, R/S). Moreover, in all combined treatments of temperature and precipitation, we compare the maximum *SC* of every functional trait to determine which trait is the most sensitive to hydrothermal change in every species.

### Statistical analysis

Values of plant morphological characteristics, biomass allocation, and growth index are subjected to an ANOVA with a General Linear Model (GLM)-Full Factorial Mode to test the main effects of warming, changing precipitation and their interaction.

A pathway analysis is chosen to analyze the main indicators affecting changes in plant biomass and the direct or indirect effects of main indicators to plant biomass. Based on a multiple regression, a pathway analysis can break the correlation coefficient *r*_*iy*_ up into direct path coefficient (direct effect of independent variable to dependent variable) and indirect path coefficient (indirect effect of independent variable to dependent variable). Firstly the main indicators affecting changes in plant biomass are filtered from functional traits by a stepwise multiple linear regression analysis. Stepwise regression is a technique for choosing variables to include in a multiple regression model, which not only guarantees the validity and importance of the chosen variables but also reduces additional error introduced by the redundant variables. In stepwise regression, the variables ending up in the final equation represent the best combination of independent variables to predict the dependent variable. In this study, we make PH, LA, LN, SLA, LAR, and R/S as independent variable *x*_*m*_ and TB as dependent variable *y*. Independent variable *x*_*m*_ and dependent variable *y* are analyzed by stepwise multiple linear regression analysis to filter the main factors *x*_*i*_ affecting dependent variable *y*. Then for independent variable *x*_*i*_ and dependent variable *y*, pathway analysis defines the indirect path coefficient (*Q*_*iy*_) between *x*_*i*_ and *y* is calculated as:

(10) $$Q_{iy}=\sum_{j=2}^{n}Q_{ijy}=\sum_{j=\;2}^{n}\left(r_{ij}\times P_{jy}\right)$$

(11) $$\begin{array}{rcl} r_{iy} & = & P_{iy}+\sum_{i\;=\;1}^{n}Q_{iy} \end{array}$$

Where *Q*_*iy*_ is indirect path coefficient of some independent variable *x*_*i*_ to dependent variable *y*; *Q*_*ijy*_ is indirect path coefficient of some independent variable *x*_*i*_ to dependent variable *y* via independent variable *x*_*j*_. *P*_*jy*_ is direct path coefficient between variable *x*_*j*_ and dependent variable *y*, and *P*_*iy*_ is direct path coefficient between variable *x*_*i*_ and dependent variable *y*, which are standardized coefficients in linear stepwise regression. *r*_*ij*_ is simple correlation coefficient between variable *x*_*i*_ and variable *x*_*j*_, and *r*_*iy*_ is simple correlation coefficient between independent variable *x*_*i*_ and dependent variable *y*, which are computed by Pearson correlation analysis.

A multiple regression analysis is used to study the correlation of plant functional traits with their local temperature and precipitation for the four *Stipa* species. For one of the species, the average temperature and precipitation data from June to August in seed collection zone are used as independent variable and data of PH, LA, LN, AB, BB, TB, SLA, LAR, and R/S are used as dependent variable. The correlation between functional traits and temperature and precipitation and goodness of fit of multiple regressions are determined by adjusted *R* square. All statistical analyses are performed using SPSS 17.0 software (SPSS Inc., Chicago, IL, USA). All statistical significances were denoted at *P* < 0.05 unless otherwise noted.

## Results

### Sensitivity of plant functional traits to changing precipitation

Changing temperature and precipitation alone significantly affected all the morphological characteristics and biomasses in the four *Stipa* species. However, the growth indices were affected differently (Table 2). For *S. grandis* and *S. bungeana*, changing temperature and precipitation significantly exerted interactive effects on every functional trait except for LN and PH (*P* = 0.100 and 0.242, *df* = 16). Functional traits of *S. breviflora* were also significantly affected by the interactive effects of changing temperature and precipitation except for AB, LN and LAR (*P* = 0.242, 0.178, and 0.226, *df* = 16). In addition, significant interactive effects occurred only in BB, TB, R/S and LAR of *S. baicalensis* (*P*-values were 0.000, *df* = 16, Table 2).

**Table 2:** **Analysis of variance for ***Stipa*** species under different temperature and precipitation treatments**.

**Source**		**Morphological characteristics**			**Biomass**			**Growth index**		
		**PH**	**LN**	**LA**	**AB**	**BB**	**TB**	**R/S**	**SLA**	**LAR**
*S. baicalensis*	T	4.19^**^	4.15^**^	1.38	1.46	2.73^*^	3.75^**^	1.09	4.63^**^	6.17^**^
	P	2.45^*^	0.72	3.57^**^	10.64^**^	11.22^**^	16.25^**^	4.46^**^	1.44	6.52^**^
	T × P	1.57	1.21	1.20	1.66	6.73^**^	8.27^**^	3.23^**^	1.47	6.22^**^
*S. grandis*	T	8.73^**^	3.08^*^	28.96^**^	40.78^**^	29.83^**^	54.42^**^	1.77	2.55^*^	3.87^**^
	P	4.54^**^	9.21^**^	11.20^**^	21.00^**^	36.43^**^	51.35^**^	4.29^**^	0.74	3.56^*^
	T × P	4.27^**^	2.02^**^	4.18^**^	7.18^**^	4.03^**^	6.32^**^	2.82^**^	2.73^**^	3.47^**^
*S. breviflora*	T	10.03^**^	6.54^**^	7.31^**^	8.25^**^	9.86^**^	9.73^**^	11.39^**^	4.30^**^	0.51
	P	18.12^**^	9.52^**^	3.75^**^	20.29^**^	10.65^**^	18.91^**^	1.84	5.45^**^	4.39^**^
	T × P	2.43^**^	1.35	4.45^**^	1.29	3.15^**^	3.20^**^	3.02^**^	2.57^**^	1.31
*S. bungeana*	T	5.93^**^	20.34^**^	18.68^**^	4.41^**^	17.40^**^	14.05^**^	13.48^**^	2.41	3.38^*^
	P	14.74^**^	17.51^**^	30.89^**^	46.50^**^	11.51^**^	32.02^**^	5.36^**^	9.25^**^	3.63^*^
	T × P	1.25	1.54	7.15^**^	4.67^**^	8.29^**^	9.69^**^	4.36^**^	3.04^**^	4.91^**^

*F-values in ANOVA for temperature, precipitation and their interaction on plant functional traits. T, Temperature; P, Precipitation; PH, Plant height; LN, The number of leaves; LA, Leaf area; AB, Aboveground biomass; BB, Belowground biomass; TB, Total biomass; R/S, Root shoot ratio; SLA, Specific leaf area; LAR, Leaf mass ratio*.

* Significant value: P < 0.05;

** *Significant value: P < 0.01*.

**Figure 1:**
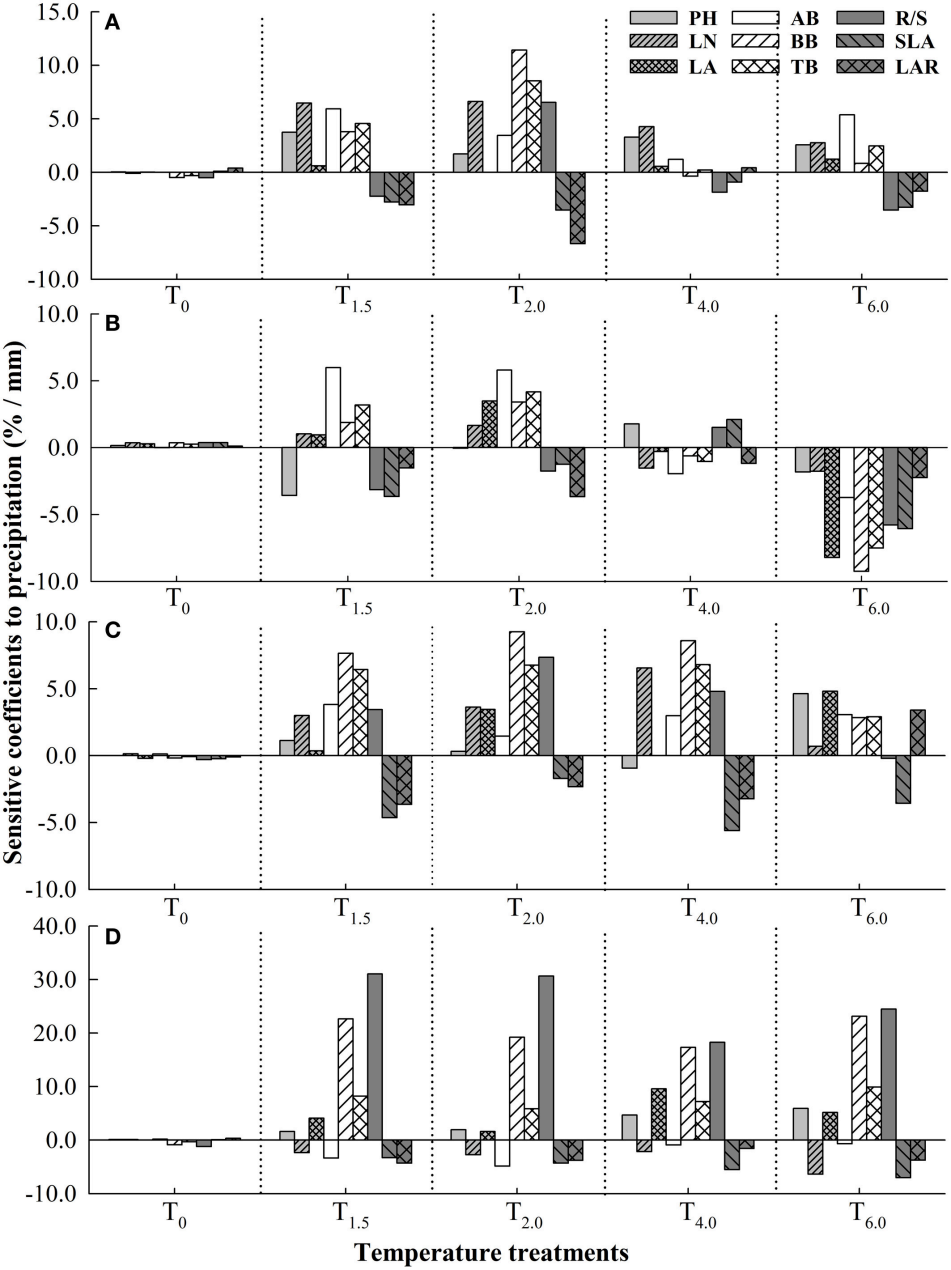
**Sensitivity coefficients of plant functional traits to precipitation under different temperature treatments in four ***Stipa*** species. (A)** *S. baicalensis*, **(B)** *S. grandis*, **(C)** *S. breviflora*, **(D)** *S. bungeana*. T_0_, T_1.5_, T_2.0_, T_4.0_, T_6.0_ denote increasing temperature by 0°C, 1.5°C, 2.0°C, 4.0°C, and 6.0°C, respectively, relative to mean temperature at the local site over 30 years (1978–2007).

Under different temperature treatments, sensitivity coefficients of plant functional traits to precipitation change were different in the four *Stipa* species. Compared to T_0_, increased temperature enhanced the sensitivity of functional traits to precipitation, but excessively high temperature reduced the sensitivity (Figure 1). For *S. baicalensis*, as temperature increased, PH, LN and AB all reached the maximum sensitivity to precipitation under T_1.5_ treatment, but LA and the other functional traits (BB, TB, SLA, LAR and R/S) reached the maximum *SC* respectively under T_6.0_ treatment and T_2.0_ treatment. For *S. grandis*, when temperature increased at T_6.0_, every functional trait (except for PH, AB, and LAR) had larger *SC* to precipitation than under the other temperature treatments. For *S. breviflora*, the traits that were the most sensitive to precipitation were LA and BB respectively under T_6.0_ and the other increased temperature treatments (T_1.5_, T_2.0_, T_4.0_). For *S. bungeana*, R/S had the maximum *SC* to precipitation under all temperature treatments (Figure 1).

Under T_0_ treatment, all the four species had low sensitivity to precipitation. As temperature increased, *S. bungeana* had larger sensitivity to precipitation than the other three species, but *S. grandis* was the most non-sensitive one among the four species. *S. breviflora* was similarly sensitive to precipitation with *S. baicalensis* when temperature increased slightly (T_1.5_, T_2.0_) and was more susceptible to precipitation than *S. baicalensis* as temperature increased largely (T_4.0_, T_6.0_; Table 3).

**Table 3:** **Average sensitivity coefficients of functional traits to precipitation under different temperature treatments (%/mm) and average sensitivity coefficients of functional traits to temperature under different precipitation treatments (%/°C) in the four ***Stipa*** species**.

**Sensitive**	**Species**			
**coefficients**	***S. baicalensis***	***S. grandis***	***S. breviflora***	***S. bungeana***
**TEMPERATURE TREATMENTS**				
T_0_	−0.10	0.25	−0.09	−0.18
T_1.5_	1.89	0.12	1.95	6.04
T_2.0_	3.12	1.31	3.13	4.85
T_4.0_	0.76	−0.14	2.22	5.21
T_6.0_	0.74	−5.16	2.06	5.64
**PRECIPITATION TREATMENTS**				
W_−30%_	−0.46	−9.59	−1.54	3.15
W_−15%_	6.32	−6.97	−0.07	9.45
W_0_	3.21	−0.45	3.62	8.44
W_+15%_	0.23	1.44	1.57	11.95
W_+30%_	−1.03	4.24	−1.28	3.73

### Sensitivity of plant functional traits to changing temperature

**Figure 2:**
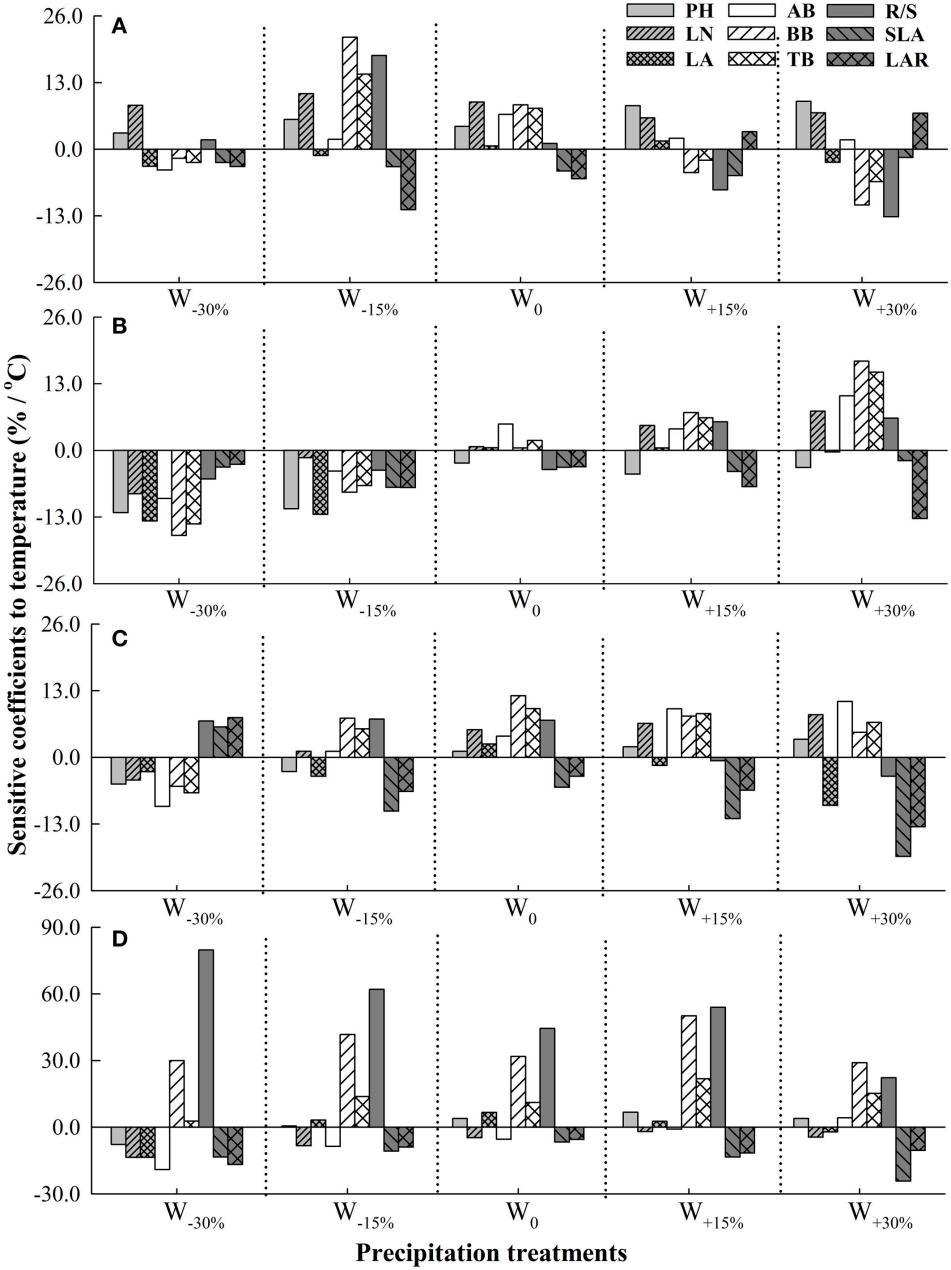
**Sensitivity coefficients of plant functional traits to temperature under different precipitation treatments in four ***Stipa*** species. (A)** *S. baicalensis*, **(B)** *S. grandis*, **(C)** *S. breviflora*, **(D)** *S. bungeana*. W_−30%_, W_−15%_, W_0_, W_+15%_, W_+30%_ denote −30%, −15%, 0, +15%, and +30% of precipitation relative to mean precipitation in the local site over 30 years (1978–2007).

Increased or decreased precipitation with a certain degree strengthened sensitivity of plant functional traits to changing temperature in the four *Stipa* species (Figure 2). Under W_0_, LN, AB, BB, and R/S were respectively the most sensitive trait to changing temperature in *S. baicalensis, S. grandis, S. breviflora*, and *S. bungeana*. Changes in precipitation altered the sensitivity of plant functional traits to temperature. For *S. baicalensis*, R/S and LN respectively had the most sensitivity to temperature when precipitation increased and decreased greatly (W_+30%_ and W_−30%_). For *S. grandis* and *S. breviflora*, BB and SLA respectively were most susceptible to temperature under increased precipitation treatments. For *S. bungeana*, R/S was the most sensitive trait to temperature when precipitation decreased or increased slightly, and BB had the maximum *SC* when precipitation significantly increased (Figure 2).

Under all precipitation treatments except for W_+30%_ treatment, *S. bungeana* was the most sensitive one to temperature among the four species. Under W_0_, sensitivity of the four species to temperature was ranked as: *S. bungeana* > *S. breviflora* > *S. baicalensis* > *S. grandis*. As precipitation deceased, *S. bungeana* had the most sensitivity and *S. grandis* was the most non-sensitivity to temperature. But *S. baicalensis* had the minimum *SC* to temperature compared to other three species as precipitation increased (Table 3).

### Sensitivity of *Stipa* species

**Figure 3:**
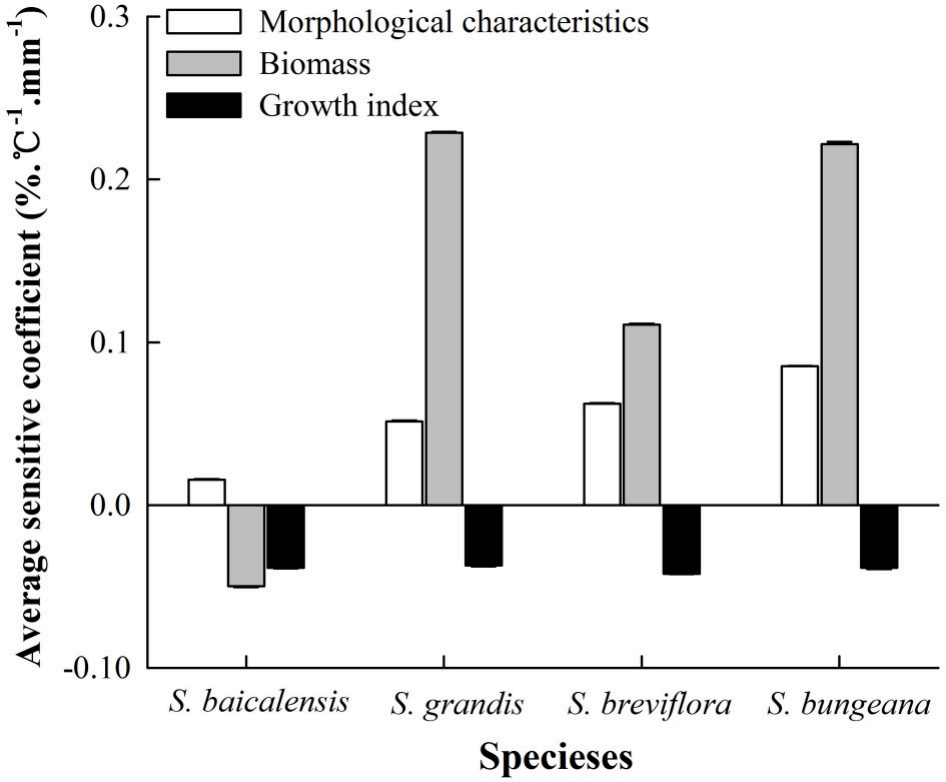
**Average sensitivity coefficients of functional traits to combined effects of changing temperature and precipitation in the four ***Stipa*** species**.

For all combined treatments of temperature and precipitation, the sensitivities of biomass in the four *Stipa* species were the maximum among all plant functional traits. The growth index was more sensitive than morphological characteristics to hydrothermal changes in *S. baicalensis*, but the opposite result was observed in the other three species (Figure 3). Compared to control hydrothermal treatment (T_0_W_0_), the average *SC* of all functional traits in the four species to hydrothermal change was ranked as: *S. bungeana* (0.09) > *S. grandis* (0.08) > *S. breviflora* (0.04) > *S. baicalensis* (−0.02).

Based on adjusted *R*^2^ in multiple regression models of plant functional traits with their local average temperature and precipitation, a faint relation of functional traits with temperature and precipitation was shown in *S. baicalensis* (Table 4). The correlation of LN, LA, AB, and R/S with temperature and precipitation in the four species was ranked as: *S. bungeana* > *S. grandis* > *S. breviflora*. *S. grandis* represented the maximum correlation of BB, TB and LAR with temperature and precipitation among the four species (Table 4). In general, *S. bungeana* and *S. grandis* exerted relatively stronger correlation with their local temperature and precipitation compared to *S. breviflora* and *S. baicalensis*, which were similar with their sensitivity degrees. Thus, the sensitivity of *Stipa* species to hydrothermal change was closely related to the hydrothermal environment in their native habitats.

**Table 4:** **Multiple regression analyses for plant functional traits with average temperature and precipitation data from June to August in seed collection zone of four ***Stipa*** species**.

	***S. baicalensis***		***S. grandis***		***S. breviflora***		***S. bungeana***	
	***R*^2^**	***P***	***R*^2^**	***P***	***R*^2^**	***P***	***R*^2^**	***P***
PH	0.05	0.010	0.02	0.102	0.29	0.000	0.27	0.000
LN	0.01	0.783	0.16	0.000	0.14	0.000	0.44	0.000
LA	0.02	0.068	0.19	0.000	0.09	0.000	0.20	0.000
AB	0.08	0.012	0.43	0.000	0.51	0.000	0.55	0.000
BB	0.00	0.421	0.56	0.000	0.16	0.001	0.18	0.000
TB	0.01	0.672	0.59	0.000	0.31	0.000	0.34	0.000
R/S	0.04	0.070	0.06	0.045	0.02	0.745	0.12	0.004
SLA	0.02	0.198	0.02	0.166	0.13	0.002	0.16	0.000
LAR	0.12	0.169	0.19	0.001	0.08	0.022	0.01	0.593

*Adjusted R Square and P-values of multiple regression models were shown*.

### Sensitivity indicators

**Figure 4:**
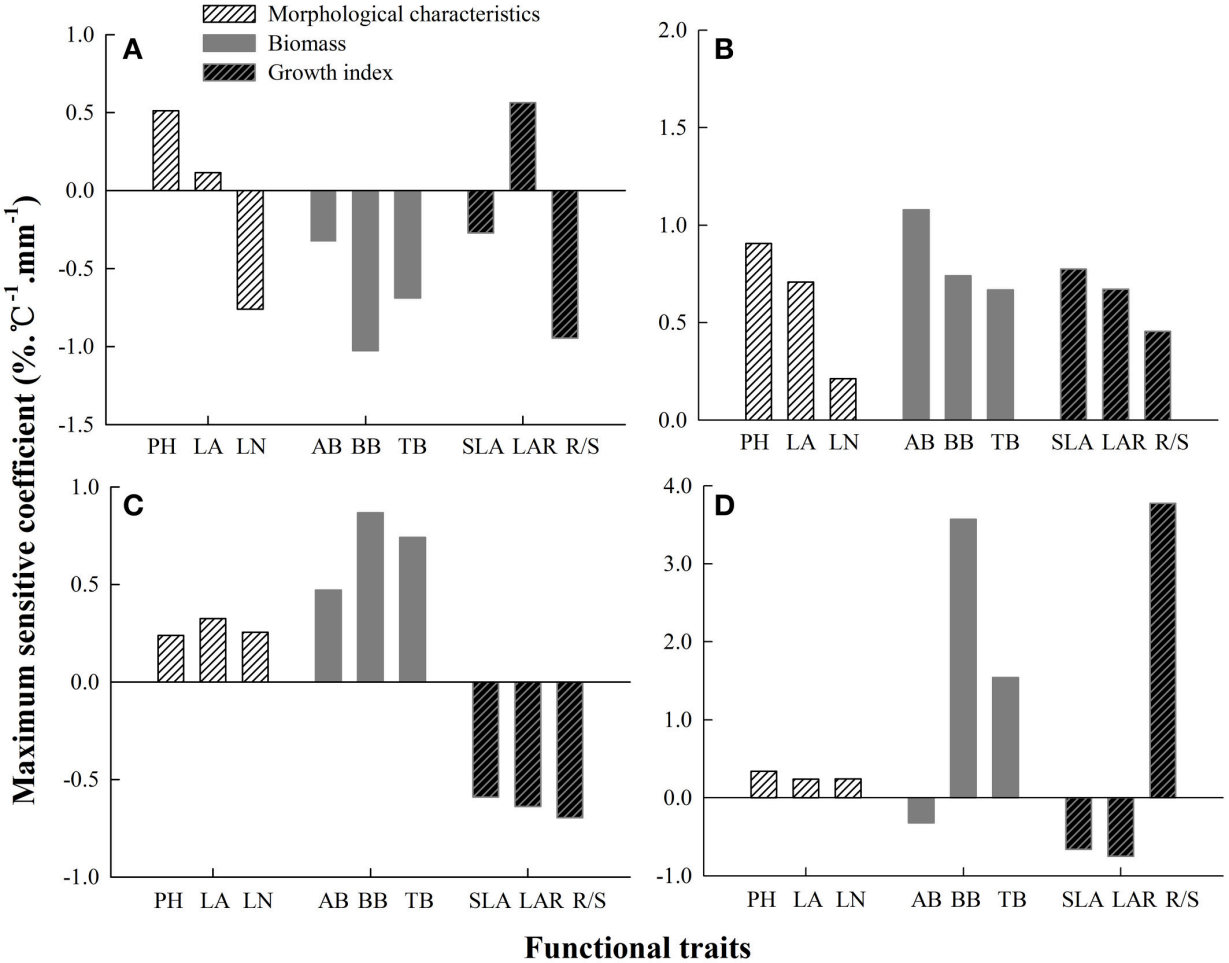
**The maximum coefficients of the sensitivity of plant functional traits to the combined effects of changing precipitation and temperature in four ***Stipa*** species. (A)** *S. baicalensis*, **(B)** *S. grandis*, **(C)** *S. breviflora*, **(D)** *S. bungeana*.

Every functional trait had different sensitivity under different combined effects of temperature and precipitation. For *S. baicalensis*, the maximum *SC* of morphological characteristics, biomass and growth indices to hydrothermal changes were LN, BB, and R/S ratio, respectively (Figure 4A). LN, BB, and R/S showed significant correlations with each other, and the correlation coefficients were −0.49 (LN and BB, *P* = 0.000, *n* = 300), 0.28 (LN and R/S, *P* = 0.000, *n* = 300), and 0.13 (BB and R/S, *P* = 0.017, *n* = 300; Table 5). The sensitivity of the three traits was BB > R/S > LN. This reflected the fact that production capacity and adaptive strategy were significantly changed by changes in hydrothermal conditions; production capacity was the most sensitive.

**Table 5:** **Correlation analysis between different plant functional traits of the four ***Stipa*** species**.

**Correlation coefficients**	**LN**	**LA**	**AB**	**BB**	**R/S**	**SLA**	**LAR**
PH	−0.59^**^	0.09	0.26^**^	0.06	0.27^**^	−0.22^**^	−0.37^**^
LN		0.55^**^	0.44^**^	−0.49^**^	0.28^**^	0.32^**^	0.45^**^
LA			0.79^**^	−0.58^**^	−0.06	0.44^**^	0.45^**^
AB				−0.60^**^	0.21^**^	−0.05	0.06
BB					0.13^*^	−0.11^**^	−0.22^**^
R/S						0.09	−0.49^**^
SLA							0.69^**^

* P < 0.05;

** *P < 0.01*.

For *S. grandis*, PH, AB, and SLA were the most sensitive traits of morphological characteristics, biomass and growth indices to hydrothermal changes, respectively (Figure 4B). The sensitivity of the three traits was AB > PH > SLA. PH had significant correlation coefficient with AB (0.26) and SLA (−0.22). But AB was not significantly correlated with SLA (*P* = 0.216, *n* = 300, Table 5). Therefore, the most sensitive indicators of hydrothermal change were AB and SLA, which reflecting plants production capacity and the latter reflected adaptive strategy to the environmental changes.

For *S. breviflora*, the most sensitive traits of morphological characteristics, biomass and growth indices were LA, BB and R/S (Figure 4C). Their sensitivities were BB > R/S > LA. BB had significant correlation coefficients with LA (−0.58) and R/S (0.13). Production capacity and adaptive strategy were sensitive to hydrothermal changes, but production capacity was the most sensitive.

For *S. bungeana*, PH, BB, and R/S were the most sensitive traits of morphological characteristics, biomass and growth indices (Figure 4D). R/S was more sensitive than PH and BB. In addition, R/S was significantly correlated with PH (0.27) and BB (0.13, Table 5). Therefore, adaptive strategy (R/S) was most sensitive to hydrothermal change.

### Screening in indicating factors of biomass

Based on a stepwise regression analysis, LA, SLA, and R/S accounted for 86% of the variance in plant biomass of the four *Stipa* species. A pathway analysis showed that of the three factors, R/S had the maximum correlation coefficient with plant biomass in *S. baicalensis* (0.82) and *S. breviflora* (0.54), as well as LA in *S. bungeana* (0.58) and *S. grandis* (0.62). SLA had the maximum indirect path coefficient with plant biomass in the four *Stipa* species (Table 6). Additionally, LA, SLA, and R/S were correlated with the other plant functional traits (Table 5), and R/S was not significantly correlated with SLA and LA (*P* = 0.066 and 0.181, *n* = 300). Thus, LA, SLA, and R/S were representative and indicative for plant growth in the four *Stipa* species.

**Table 6:** **Pathway analyses for main factors influencing plant biomass in the four ***Stipa*** species (— indicates none)**.

**Species**	**Independent variable**	**Correlation coefficient**	**Direct path coefficient**	**Indirect path coefficient**			
				**LA**	**SLA**	**R/S**	**Total**
*S. baicalensis*	LA	0.283	0.276	—	−0.070	0.077	0.007
	SLA	−0.065	−0.335	0.058	—	0.213	0.270
	R/S	0.815	0.872	0.024	−0.082	—	0.057
*S. grandis*	LA	0.623	0.898	—	−0.286	0.010	0.276
	SLA	−0.196	−0.735	0.349	—	0.190	0.540
	R/S	0.183	0.464	0.020	−0.301	—	0.282
*S. breviflora*	LA	0.339	0.779	—	−0.550	0.107	0.443
	SLA	−0.212	−0.890	0.481	—	0.188	0.670
	R/S	0.541	0.667	0.125	−0.251	—	0.126
*S. bungeana*	LA	0.584	0.819	—	−0.191	−0.044	0.235
	SLA	−0.291	−0.600	0.261	—	0.047	0.309
	R/S	0.339	0.474	−0.075	−0.060	—	0.135

Combined with the maximum sensitivity of plant functional traits to hydrothermal change (Figure 4), the most sensitive factors indicating plant biomass were adaptive strategy (R/S) in *S. baicalensis* and *S. bungeana*, the instantaneous environmental effect (SLA) in *S. grandis*, and production capacity (LA) and adaptive strategy (R/S) in *S. breviflora*.

## Discussion

Because of differences in regional climate change and each species' biological characteristics, there are remarkable species-specific responses to changing temperature and precipitation (Donovan et al., 2011; Xu et al., 2014; Mowll et al., 2015). In this study, the responses of plant functional traits to combined effects of temperature and precipitation were determined for dominant species from the grasslands of Inner Mongolia. Our findings were consistent with previous results (Xu et al., 2014; Maréchaux et al., 2015); the four *Stipa* species had their own individual response sensitivities to hydrothermal changes (Figure 3). Of these species, *S. baicalensis* mainly distributes in semi-arid and sub-humid meadow steppe with a relatively sufficient water supply (Qi et al., 2010). Compared to *S. bungeana, S. breviflora*, and *S. grandis*, the correlation of plant functional traits with average temperature and precipitation of the growing season was relatively weaker in *S. baicalensis* (Table 4). Simultaneously *S. baicalensis* was the least sensitive to hydrothermal changes with an average sensitivity coefficient of −0.2, which reflected the fact that future climate change may had a little negative effect on *S. baicalensis*. *S. bungeana* was the dominant species in warm-temperate typical steppe (Hu et al., 2013) with the relatively high demand of combined temperature and precipitation conditions (Table 4). Thus, the sensitivity of *S. bungeana* was the strongest among the four *Stipa* species (Figure 3). *S. grandis* was most suitable to grow in the unique environment of typical grassland. In addition, it would be replaced by *S. baicalensis* if the habitat conditions tended to become more mesic and by *S. krylovii* when environmental conditions tended to be more drought prone (Qi et al., 2010). *S. breviflora* mainly occurs in arid and semi-arid desert steppe and has a well-developed root system and higher drought resistance (Hu et al., 2014). Additionally, plant biomass of *S. grandis* had higher correlation with temperature and precipitation than *S. breviflora* (Table 4). Therefore, *S. grandis* was more vulnerable than *S. breviflora* in response to combined effects of temperature and precipitation (Figure 3).

Under the background of climate change, changes in temperature and precipitation are simultaneous and make combined effects on plants (IPCC, 2013; Mora et al., 2013). Numerous studies have reported that slight warming could promote plant cell division and growth, and then promote an increased photosynthetic rate and plant growth (Bret-Harte et al., 2001). However, greater warming would increase evaporation (Springate and Kover, 2014), then this would further weaken the plant's supply of precipitation and so inhibit plant growth (Xu and Zhou, 2006). Therefore, the response of plants to precipitation could be altered by changing temperature, and vice versa (Qaderi et al., 2012; Xu et al., 2014). In this study, sensitivity of plant functional traits in response to changes in precipitation was strengthened by increased temperature in a certain degree (Figure 1). Additionally, sensitivities of plant functional traits to temperature were also shifted by changing precipitation (Figure 2). For example, for *S. baicalensis*, BB was the most susceptible trait to temperature among plant functional traits under slightly decreased precipitation (W_−15%_), but LN had the maximum *SC* to temperature under an excessive shortage of precipitation (W_−30%_, Figure 2). This reflected the fact that as precipitation slightly reduced, *S. baicalensis* might mainly increase root cell division significantly and allocate more sources to roots to response to temperature change (Bret-Harte et al., 2001). While under drought stress, this species might choose to decrease leaf cell volume/surface area and increase LN to adapt to temperature change (Schäffner, 1998).

Plant individual biomass is the final result of photosynthesis and stands at the core of the grassland research (Ehret et al., 2015), which is not only restricted by the plant's own biological characteristics, but also greatly affected by environmental factors (Myneni et al., 1997; Zhang et al., 2011). Because plant biomass is not easy to measure, it is essential to screen various indicators which can reflect changes in plant biomass and are sensitive to environmental changes among plant functional traits. These chosen indicators are important for evaluating and predicting the response and adaptation of *Stipa* species and grassland ecosystems to climate change. In this study, LA, R/S, and SLA accounted for 86% of the variance in plant biomass in the four *Stipa* species. Moreover, these three traits had some direct and indirect correlations with plant biomass (Table 6), and they were also related with the other functional traits (Table 5). To some extent, they reflected plant production capacity (LA), adaptive strategy (R/S), instantaneous environment effects (SLA) and cumulative environmental effects (LA and R/S; Poorter, 1999; Yang et al., 2010). This partly confirmed the previous results that plant leaf and root mass partitioning were more significantly affected by temperature and precipitation (Bret-Harte et al., 2001; Catoni and Gratani, 2014; Xu et al., 2014; Salgado-Negret et al., 2015). Thus, these three traits could be used as sensitive indicators to hydrothermal change in the four *Stipa* species analyzed here. The most sensitive indicators representing plant biomass to hydrothermal change were adaptive strategy (R/S) in *S. baicalensis* and *S. bungeana*, leaf adaptive strategy (SLA) in *S. grandis*, and the cumulative environmental effects (LA and R/S) in *S. breviflora* (Figure 4; Table 6). This suggested that the responses of plants to hydrothermal change could not be reflected by a single indicator, but a combination of multiple indicators was needed. The responses of plants to environmental factors are complicated, so screening sensitive indicators of plants in response to environmental factors (temperature, precipitation, CO_2_, etc.) and estimating their adaptive thresholds require further study.

## Conclusions

Based on combined effects of temperature and precipitation, the three hypotheses were partly confirmed. Increased temperature to a certain degree enhanced the sensitivity of plant functional traits in response to precipitation, and vice versa. The sensitivity of the four *Stipa* species with specific traits to changing temperature and precipitation varied. The sensitive indicators to hydrothermal changes were LA, SLA, and R/S in the four *Stipa* species. This suggests that the sensitivity of *Stipa* plants to hydrothermal changes should be reflected by a combination of multiple indicators. Also the responses of sensitive indicators can play a crucial role in the adaptation of *Stipa* species to future climate change.

## Author contributions

GZ, YW, and XL conceived and designed the research. XL, YW, and XS conducted the experiment. All authors analyzed and interpreted the data. XL and GZ wrote the manuscript; all authors discussed and approved the final version.

## Funding

This study was jointly funded by Chinese Academy of Sciences “Strategic Priority Research Program-Climate Change: Carbon Budget and Relevant Issues” (XDA-05050408) and China Special Fund for Meteorological Research in the Public Interest (Major projects) (GYHY201506001-3).

### Conflict of interest statement

The authors declare that the research was conducted in the absence of any commercial or financial relationships that could be construed as a potential conflict of interest. The Associate Editor Jian-Guo Huang declares that, despite being affiliated with the same institute as the authors Xiaomin Lv, Guangsheng Zhou, Yuhui Wang and Xiliang Song, the review process was handled objectively.

## Abbreviations

PH: Plant height
LN: The number of leaves
LA: Leaf area
AB: Aboveground biomass
BB: Belowground biomass
TB: Total biomass
R/S: Root/shoot ratio
SLA: Specific leaf area
LAR: Leaf mass ratio
*SC*: Sensitivity coefficient.
